# Genome Identification, Expression Profile Analysis, and Abiotic Stress Response Mechanism of Longan *BES1* Gene

**DOI:** 10.3390/ijms26073003

**Published:** 2025-03-25

**Authors:** Zilu Zeng, Ronglin Liu, Jin Zhao, Shuoxian Lan, Hao Yang, Hua Wu, Yuling Lin, Shijiang Cao

**Affiliations:** 1 College of Forestry, Fujian Agriculture and Forestry University, Fuzhou 350002, China; 15879367967@163.com (Z.Z.); d1315068727@163.com (R.L.); 13935362057@163.com (J.Z.); m15803366858@163.com (H.Y.); 2 Institute of Horticultural Biotechnology, Fujian Agriculture and Forestry University, Fuzhou 350002, China; lanshuoxian2023@163.com; 3 College of Food Science, Fujian Agriculture and Forestry University, Fuzhou 350002, China; w2918083657@163.com

**Keywords:** *Dimocarpus longan* Lour., *BES1* gene family, IAA response, evolutionary analysis, abiotic stress

## Abstract

BES1 (BRI1 EMS SUPPRESSOR 1) is a critical transcription factor involved in plant growth, development, and stress responses. Although *BES1* genes have been characterized in several species, their roles in longan (*Dimocarpus longan* Lour.) remain unclear. This study identified and analyzed eight *BES1* genes in the longan genome. Phylogenetic analysis classified these genes into four subgroups (I-IV), with conserved motifs and intron–exon structures indicating potential functional similarities within subgroups. Cis-element analysis revealed that the promoters of *DlBES1* genes contain numerous hormone-related elements, including ABRE, TGACG, and TCA motifs, suggesting their involvement in hormonal signaling and stress responses. Expression profiling showed differential expression patterns of *DlBES1* genes across nine tissues, with notable up-regulation in roots and seeds. Additionally, *DlBES1* genes exhibited distinct expression trends under varying temperatures and in response to IAA treatment, indicating potential roles in temperature stress adaptation and hormone signaling. These findings provide novel insights into the regulatory mechanisms of *BES1* genes in longan and highlight their potential significance in stress tolerance and growth regulation.

## 1. Introduction

Brassinosteroids (BRs) are a group of plant-specific steroid hormones that were first identified in oilseed rape. They constitute the sixth class of phytohormones, in addition to auxin, gibberellin, cytokinin, abscisic acid, and ethylene [1,2]. BRs play an important role in various aspects of plant growth, development, and reproduction, as well as in responses to high- and low-temperature stress, drought, salinity, and insect attacks [3,4,5,6]. BRs also promote cell elongation, influence vascular differentiation, coordinate cell layer interactions, participate in photomorphogenesis, stomata formation, and stem cell self-renewal and death, promote seed germination, regulate flowering and male fertility, and enhance plant stress resistance [5,7,8,9,10,11,12,13]. The BR signaling pathway can interact with several other pathways to regulate plant growth and development and respond to abiotic stresses. For instance, it interacts with abscisic acid (ABA) signaling to enhance plant resistance to abiotic stress [14]. Additionally, the BR signaling pathway interacts with the drought response through RD26, exerting a negative regulatory effect on the plant’s response to drought [15]. Other studies have shown that the BR signaling pathway also crosstalks with other hormone signaling pathways such as auxin [16], ethylene (ET), jasmonic acid (JA) [4], salicylic acid (SA) [17], abscisic acid (ABA) [18,19], and gibberellin (GA) [20,21]. These functions of BRs are facilitated by the participation of an important transcription factor, Bri1 EMS Suppressor 1 (BES1).

**Figure 1:**
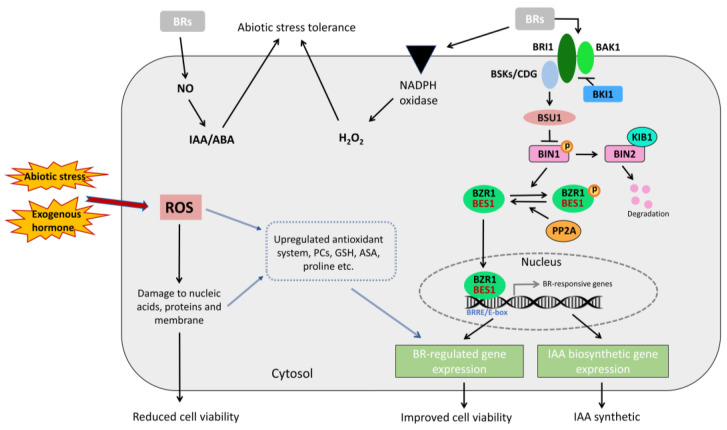
Working model of *BES1* transcription factor and schematic diagram of plant cell responses to stress (modified from Li, etc. [41]; Yamaguchi-Shinozaki, etc. [42]).

*BES1* is a prominent transcription factor in modulating the BR signaling pathway and downstream gene expression [22], as shown in Figure 1. The *BES1* gene family comprises *BES1* (also known as BZR2), BZR1, and four homologs named BEH1, BEH2, BEH3, and BEH4 [23,24]. BES1 has an atypical basic helix–loop–helix (bHLH) domain, which can specifically bind to the E-box (CANNTG) or the BR response element (BRRE, CGTG(T/C)G), thereby directly activating or inhibiting downstream gene expression [25,26]. The mechanism by which the BES1 transcription factor regulates the BR signaling pathway has been elucidated. The BR signaling pathway comprises well-defined components, including Brassinosteroid Insensitive 1 (BRI1), BAK1, BKI1, BSKs, BSU1, and BIN2. BRI1 is a membrane-localized leucine-rich repeat receptor-like kinase (LRR-RLK) containing 25 leucine-rich repeats (LRRs) and an island domain that binds to BR [27,28,29,30]. BRI1 has three homologs, BRL1, BRL2, and BRL3, but only BRL1 and BRL3 encode BR receptors [31]. BAK1 is another leucine-rich repeat-rich serine/threonine-type receptor-like kinase that forms a heterodimer with BRI1 to transmit BR signals [32,33]. When BR binds to BRI1, it initially activates BRI1 to a basal level, which then binds to BAK1 and activates it [34,35]. Activated BAK1 phosphorylates BRI1, enhancing BRI1 kinase activity and substrate phosphorylation, leading to the amplification of BR signaling [33,34,36]. BKI1 is a plasma membrane-bound phosphorylated protein that directly interacts with the kinase domain of BRI1 to interfere with BRI1 activation [37]. BSKs are substrates of BRI1 kinases and include three homologous BR-signaling kinases: BSK1, BSK2, and BSK3 [38]. BSU1 is a nuclear-localized phosphatase with an N-terminal Kelch-repeat domain [39]. BIN2 is a negative regulator in the BR signaling pathway, phosphorylating BES1 and BZR1 to inhibit their activity [39,40].

When BR is absent, phosphorylated *BES1* and *BZR1* recruit and bind to 14-3-3 proteins and remain in the cytoplasm, preventing these transcription factors from entering the nucleus to bind to BR-responsive genes [43]. In the presence of BR, BKI1 dissociates from the membrane. Isolated BKI1 is not directly degraded but enhances BR signaling by interacting with the 14-3-3 protein [44,45]. BRI1 phosphorylates BSK. Phosphorylated BSK dissociates from BRI1 and activates BSU1 phosphatase. Activated BSU1 inhibits BIN2 by dephosphorylating it at Tyr200, dephosphorylating *BES1* and *BZR1* [40,46]. Consequently, *BES1* and *BZR1* can enter the nucleus and bind to the promoters of BR target genes to regulate their expression [47]. Protein Phosphatase 2A (PP2A) additionally enhances BR signaling by dephosphorylating *BZR1* and *BES1*, thereby exerting a positive regulatory effect [38,48,49].

Longan is one of the most important subtropical fruit trees in the world [50]. *BES1*, an important hormone-related transcription factor, has been identified and analyzed in many species, such as *Arabidopsis thaliana* (L.) [51], *Oryza sativa* L. [52], and *Piper nigrum* L. [53]. We conducted whole-genome identification and analysis of the *BES1* genes in longan. In our study, we analyzed the physicochemical properties, evolutionary tree, gene structure, conserved structural domains, and intraspecific and interspecific collinear relationships of the longan *BES1* genes. We also determined *DlBES1* in different tissues and the differences in the expression levels of *DlBES1* genes under hormone treatments and abiotic stress. These results laid a foundation for further exploring the function of the *BES1* gene family in *D. longan*.

## 2. Results

### 2.1. Identification and Physicochemical Property Analysis of DlBES1 Gene Family

It can be obtained through analysis that eight BES1 proteins were identified in longan, designated as *DlBES1.1* to *DlBES1.8* based on their chromosomal distribution (Table 1). The encoded BES1 proteins range from 202 (DlBES1.6) to 1178 (DlBES1.8) amino acids in length, with protein size typically reflecting the length of their amino acid sequences. In this study, the relative molecular weights of the eight DlBES1 proteins varied from 21,720.31 Da (DlBES1.6) to 130,421.6 Da (DlBES1.8). Among them, DlBES1.2 and DlBES1.7 showed acidic profiles (pI < 7.0), while the remaining proteins showed alkaline profiles. Moreover, all DlBES1 proteins, except for DlBES1.7, were unstable (instability index > 40), with a range of 41.64 to 65.83. The results demonstrate that the aliphatic index ranged from 54.98 to 75.98, reflecting the thermal stability of the DlBES1 proteins. The negative GRAVY values of DlBES1s indicate that all DlBES1 proteins are hydrophilic. Subcellular localization predictions indicate that six DlBES1 proteins are exclusively localized in the nucleus, while DlBES1.2 and DlBES1.7 were found in both the cytoplasm and nucleus. These findings indicate that BES1 proteins mainly function in the nucleus, and some members may be involved in the process in the cytoplasm.

### 2.2. Chromosomal Localization of DlBES1 Gene Family

**Figure 2:**
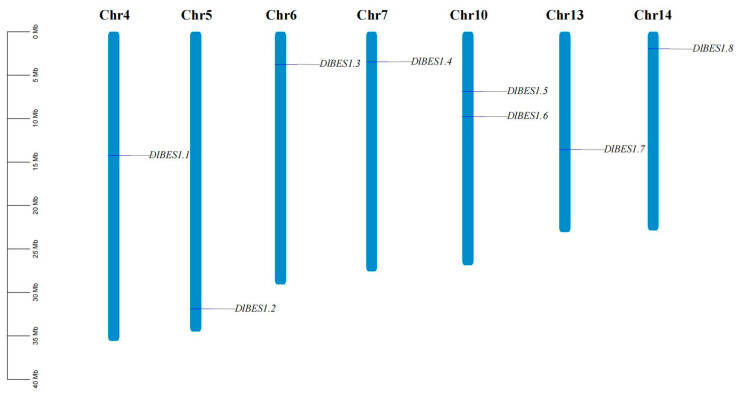
The distribution of *BES1* genes in *D. longan* chromosomes. The number of chromosomes is shown at the top of each chromosome, with their approximate size (Mb) and location on the left. The name of each *BES1* gene is annotated on the right side of the chromosome.

Chromosomal localization analysis of *BES1* genes using genomic data from *D. longan* Lour (Figure 2) showed that no BES1 gene was found on chromosomes 1, 2, 3, 8, 9, 11, and 12. Regarding the distribution of *BES1* genes on chromosomes, apart from two *BES1* genes (*BES1.5* and *BES1.6*) located on chromosome 10, each of the remaining chromosomes has only one BES1 gene distributed on it. Further analysis showed that there was no correlation between chromosome length and the number of *BES1* genes, indicating that gene distribution was not related to chromosome size.

### 2.3. Phylogenetic Analysis of the DlBES Gene Family

**Figure 3:**
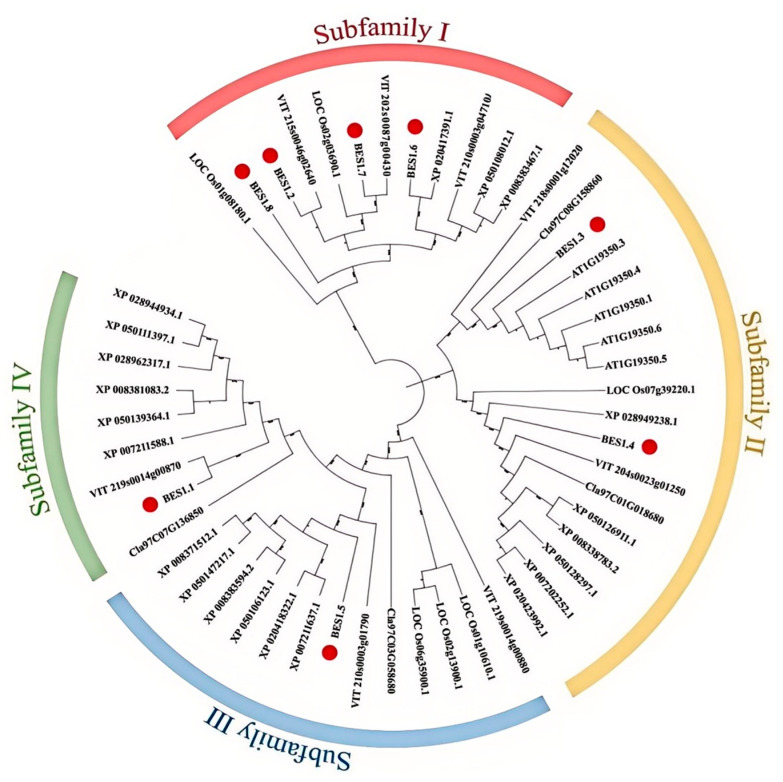
A phylogenetic tree of 6 species of BES1 proteins. The different colored arcs represent the BES1 protein subfamily. The tree was built using 8 BES1 proteins from longan (represented by the red circle), 5 BES1s from *A. thaliana* (represented by ‘AT-’), 6 BES1s from *O. sativa* (represented by ‘LOC-’), 8 BES1s from grape (represented by ‘VIT-’), 4 BES1s from watermelon (represented by ‘Cla-’), and 20 BES1s from apple (represented by ‘XP-’).

To study the phylogenetic relationships and biological functions of the *DlBES1* genes among other species, a phylogenetic tree was constructed using BES1 protein sequences from six species, including *D. longan*, *A. thaliana*, *O. sativa*, *Vitis vinifera* L., *Citrullus lanatus* (Thunb.), and *Malus pumila* Mill. (Figure 3). Analysis shows that the BES1 genes of *D. longan*, *A. thaliana*, *O. sativa*, *V. vinifera*, *C. lanatus*, and *M. pumila* are divided into four subfamilies (I–IV). The distribution of BES1 genes in these subfamilies varies from species to species; among the eight *BES1* genes identified in longan, subfamily I contains four *DlBES1* members (*DlBES1.2*, *DlBES1.6*, *DlBES1.7*, and *DlBES1.8*), while subfamily II contains two *DlBES1* members (*DlBES1.3* and *DlBES1.4*). Subfamily III and subfamily IV each contain one *DlBES1* member (*DlBES1.5* in subfamily III and *DlBES1.1* in subfamily IV).

### 2.4. Gene Structure and Conserved Motif Analysis of DlBES Gene Family

**Figure 4:**
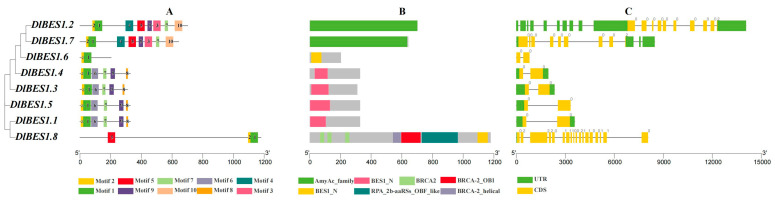
*DlBES1* gene phylogenetic relationship and gene structure schematic diagram. (**A**) The conserved motifs of *DlBES1* genes. The non-conserved sequences are presented using black lines, and 10 motifs are presented by different colored boxes numbered on the right. (**B**) The conserved domains of *DlBES1* proteins. The gene’s length can be approximated using the scale at the bottom. (**C**) Intron and exon structures of *BES1* in *D. longan*. The yellow boxes indicate exons and the black lines indicate introns, while the green boxes indicate untranslated regions (UTRs) in the upstream and downstream regions. The figure legends are shown at the bottom of each graphic.

By analyzing the conserved protein sequence motifs and intron–exon structures of eight DlBES1 gene members, we identified 10 conserved motifs. The results in Figure 4A show that motifs 1 and 2 exist in all *DlBES1* genes, indicating their vital role in *DlBES1* gene function. *DlBES1.2* and *DlBES1.7* share eight motifs, while *DlBES1.1*, *DlBES1.3*, *DlBES1.4*, and *DlBES1.5* share seven motifs. *DlBES1.6* contains the fundamental motifs (motifs 1 and 2) of the *DlBES1* gene family, while *DlBES1.8* has an additional motif (motif 5) compared to *DlBES1.6*. In terms of intron–exon analysis (Figure 4C), we found that *DlBES1.2*, *DlBES1.7*, and *DlBES1.8* have a large number of exons and introns compared to the other five DlBES1s, which only contain two exons and one intron.

### 2.5. Cis-Acting Element Analysis of DlBES1 Genes

**Figure 5:**
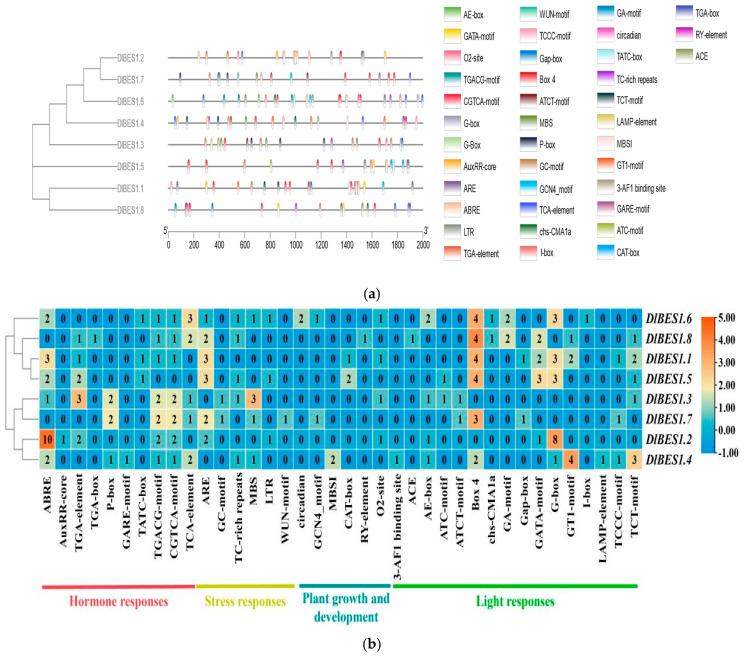
(**a**) The cis-element prediction of 8 *DlBES1* gene promoter sequences (−2000 bp) was analyzed. The cis-acting elements predicted for 2000 bp upstream of the gene are indicated on the left. Squares of different colors represent different cis-acting elements. The names of cis-acting elements and corresponding color annotations are on the right; (**b**) a heat map of 38 kinds of cis-acting elements in 8 *DlBES1* genes. The different cis-acting elements are classified into four categories: hormone response, stress response, plant growth and development, and light response. Each category is annotated in text below the heat map. The numbers in the heat map boxes indicate specific expression levels. Greater values mean higher expression, while smaller values mean lower expression.

In order to understand the biological function and regulatory network of *DlBES1* genes in longan, we detected the cis-regulatory elements in the 2000 bp promoter region upstream of *DlBES1* genes. The results reveal various cis-acting elements in *DlBES1* genes, indicating their responsibility for multiple plant functions (Figure 5). These elements are categorized into phytohormone elements, stress response elements, plant growth and development elements, and light response elements. Specifically, *DlBES1.1* contains 7 phytohormone elements, 3 stress response elements, 2 plant growth and development elements, and 15 light response elements; *DlBES1.2* contains 17 phytohormone elements, 3 stress response elements, 1 plant growth and development element, and 10 light response elements; *DlBES1.3* contains 11 phytohormone elements, 5 stress response elements, 1 plant growth and development element, and 4 light response elements; *DlBES1.4* contains 8 phytohormone elements, 2 stress response elements, 2 plant growth and development elements, and 14 light response elements; *DlBES1.5* contains 5 phytohormone elements, 5 stress response elements, 2 plant growth and development elements, and 12 light response elements; *DlBES1.6* contains 8 phytohormone elements, 4 stress response elements, 4 plant growth and development elements, and 13 light response elements; *DlBES1.7* contains 7 phytohormone elements, 5 stress response elements, 1 plant growth and development element, and 6 light response elements; *DlBES1.8* contains 6 phytohormone elements, 3 stress response elements, 1 plant growth and development element, and 12 light response elements. According to the provided results, *DlBES1.2* has the largest number of hormone response elements, followed by *DlBES1.3*. Among the *DlBES1* genes, *DlBES1.1* has the largest number of light-responsive elements, followed by *DlBES1.4* and *DlBES1.6*. Each gene has relatively fewer stress response elements and plant growth and development elements. Overall, within the *DlBES1* genes, there are a greater number of cis-acting elements related to hormone and light responses compared to stress response elements and plant growth and development elements, suggesting that *DlBES1* genes may play an essential role in *D. longan* hormone responses and are likely involved in a wide range of response processes.

### 2.6. Interspecific and Intraspecific Collinearity Analysis of DlBES1 Gene Family

**Figure 6:**
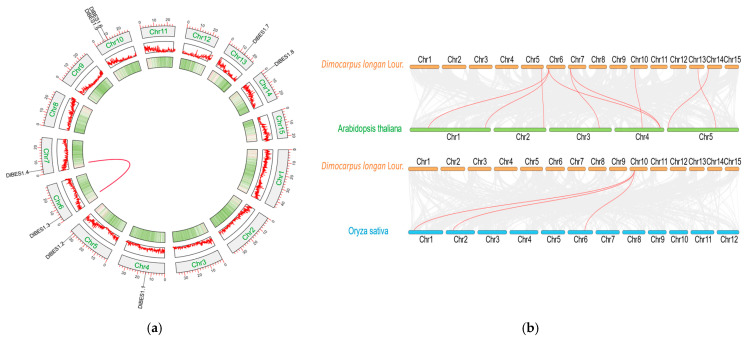
(**a**) The intraspecific collinearity of *DlBES1* genes; the red line represents the *BES1* gene pairs with a collinear relationship in the longan genome; (**b**) *D. longan*, *A. thaliana*, and *O. sativa BES1* genes interspecific collinearity analysis. The red lines highlight the syntenic gene pairs, while the gray lines in the background show the collinear blocks in the genomes of longan with *A. thaliana* and *O. sativa*.

In the interspecific collinearity analysis of *BES1*, we investigated the collinearity relationships among *A. thaliana*, *O. sativa*, and *D. longan* (Figure 6). The results demonstrate nine pairs of genes with collinearity relationships between longan and *A. thaliana* (DlBES1.2 in DlChr5 and one gene in AtChr2; DlBES1.3 in DlChr6 and two genes in AtChr1; one gene in AtChr4; DlBES1.4 in DlChr7 and AtChr3 and AtChr4; DlBES1.5 in DlChr10 and AtChr4; DlChr13 and AtChr5; DlChr14 and AtChr5) (Figure 2 and Figure 6). In the collinearity relationship between longan and *O. sativa*, only *BES1.5* in DlChr10 showed a collinearity relationship, with three genes in *O. sativa* distributed in OsChr1, OsChr2, and OsChr6. These findings suggest that *DlBES* may be more closely related to *Arabidopsis* than rice. Regarding the intraspecific collinearity relationship of *BES1* in longan, a collinearity relationship was identified between *DlBES1.3* in Chr6 and *DlBES1.4* in Chr7.

### 2.7. Analysis of Specific Expression of DlBES1 in Different Tissues, Early Developmental Stages of Somatic Embryos and Temperature

**Figure 7:**
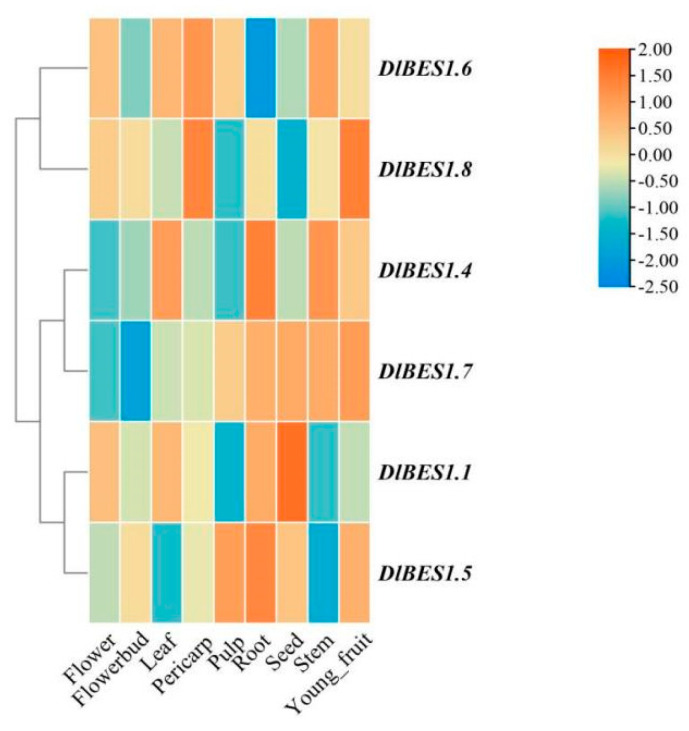
The expression profiles of DlBES1 genes in nine different tissues. The more red the box color, the higher the tissue expression, and the more blue the box color, the lower the tissue expression.

**Figure 8:**
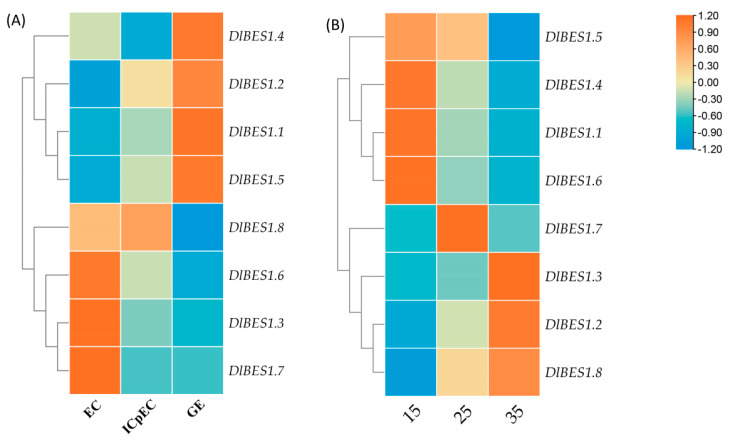
(**A**) The expression patterns of DlBES1 during different early developmental stages of somatic embryos. (**B**) The expression patterns of DlBES1 genes at 15 °C, 25 °C, and 35 °C. The higher the intensity of the red color in the box, the greater the tissue expression; conversely, a lower intensity of the blue color indicates decreased tissue expression.

The expression patterns of the *DlBES1* genes in nine tissues of longan, including flower, flower bud, leaf, pericarp, pulp, root, seed, stem, and young fruit, were compared using the longan database (Figure 7). Our results indicate that the expression of the *DlBES1* genes was generally low in flowers and buds. However, in roots and seeds, *DlBES1* gene expression was notably high compared to other tissues, except for a few genes. Among all *DlBES1* genes, *DlBES1.4* exhibited the highest expression levels in roots, stems, and leaves, while the lowest expression was observed in pulp, flowers, and flower buds. *DlBES1.7* showed high expression in roots, seeds, stems, and young fruits, with the lowest expression levels in flowers and flower buds. Additionally, *DlBES1.1*, *DlBES1.4*, *DlBES1.5*, and *DlBES1.7* exhibited high expression levels in roots but lower expression levels in flowers and flower buds. Conversely, *DlBES1.6* showed low expression levels in all nine tissues. Furthermore, we constructed an expression heat map of the *DlBES1* genes during different early developmental stages of somatic embryos (EC: embryogenic callus; ICpEC: incomplete pro-embryogenic callus; GE: globular embryo) based on the longan transcriptome database (Figure 8A). The results demonstrate that *DlBES1.4*, *DlBES1.2*, *DlBES1.1*, and *DlBES1.5* are highly expressed in the GE stage but their expression is low in the EC stage. Conversely, the expressions of *DlBES1.8*, *DlBES1.6*, *DlBES1.3*, and *DlBES1.7* are down-regulated in the development stage from EC to GE.

By analyzing the expression levels of DlBES1 genes at 15 °C, 25 °C, and 35 °C (Figure 8B), the results show that there are significant differences in the expression trend of DlBES1 genes under heat stress at different temperatures. Expressions of *DlBES1.1*, *DlBES1.4*, *DlBES1.5*, and *DlBES1.6* were highest at 15 °C and decreased with increasing temperature. In contrast, expressions of *DlBES1.2*, *DlBES1.3*, and *DlBES1.8* were lowest at 15 °C and increased with rising temperature. Although *DlBES1.7* exhibited the highest expression in the entire gene family, its expression level was not significantly affected by temperature. We speculated that expressions of *DlBES1.1*, *DlBES1.4*, *DlBES1.5*, and *DlBES1.6* may be related to longan’s resistance to low-temperature environmental stress, while the expressions of *DlBES1.2*, *DlBES1.3*, and *DlBES1.8* may be associated with longan’s resistance to heat stress. However, whether *DlBES1.7* is related to longan’s response to heat stress remains unknown.

### 2.8. Expression Patterns of D. longan DlBES1 Genes During Somatic Embryogenesis and in Response to IAA Treatment

**Figure 9:**
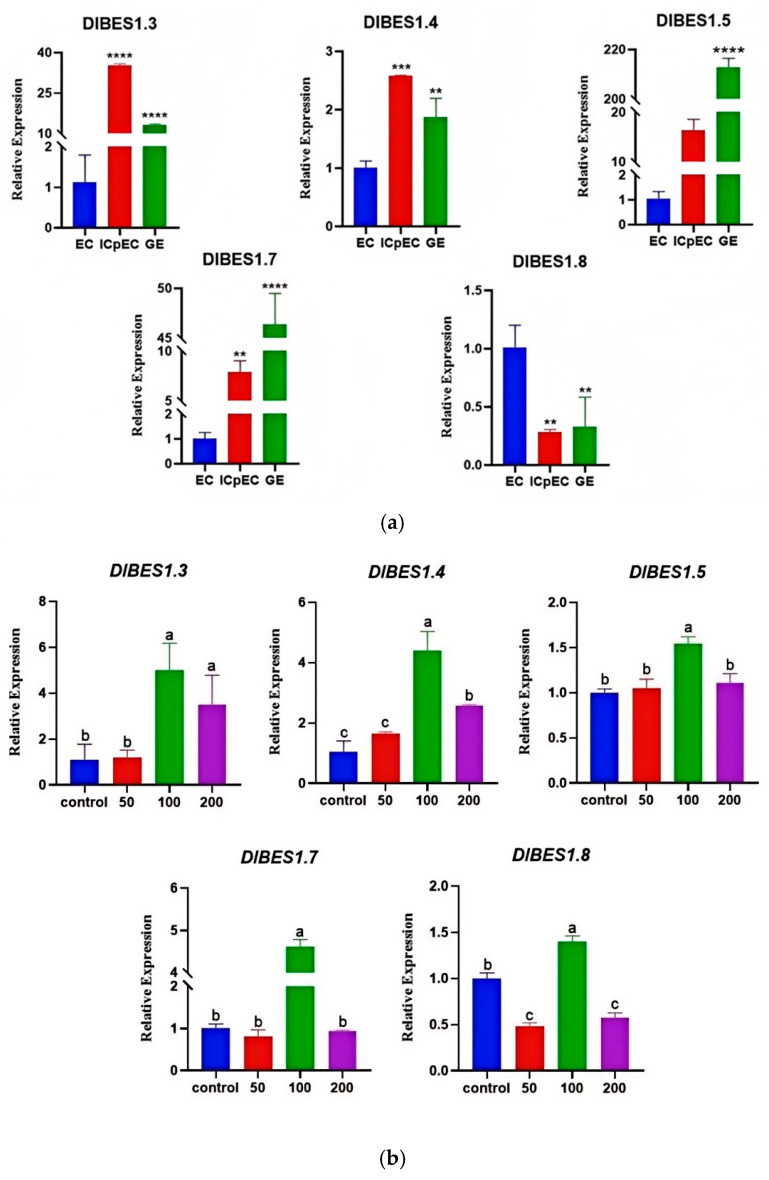
(**a**) The expression of some *DlBES1* genes during different development stages of longan early somatic embryos was analyzed using Prism 8.0.2 software. The one-way ANOVA method was employed to determine the significant difference in *DlBES1* expression during different stages of longan somatic embryos. The experiment was conducted thrice and the average value was calculated. Standard deviation was used as a statistical method to describe the extent of data dispersion. (**b**) The expression of *DlBES1* genes during embryogenic callus (EC) stage under different IAA treatments. The expression of certain *DlBES1* genes was subjected to exogenous IAA treatment at concentrations of 0 µmol·L^−1^, 50 µmol·L^−1^, 100 µmol·L^−1^, and 200 µmol·L^−1^. The identical letters on the top of the bar chart indicate no statistically significant difference, while different letters indicate a significant difference (** *p* < 0.01, *** *p* < 0.001, **** *p* < 0.0001). Note: EC: embryogenic callus; ICpEC: incomplete pro-embryogenic callus; GE: globular embryo.

By analyzing the expression of *DlBES1.3*, *DlBES1.4*, *DlBES1.5*, *DlBES1.7*, and *DlBES1.8* genes in different early stages of longan somatic embryogenesis (Figure 9a), the results show that the expression of *DlBES1.5* and *DlBES1.7* was up-regulated with the development of longan embryos, while the expression of *DlBES1.8* was down-regulated. Initially, the expression levels of *DlBES1.3* and *DlBES1.4* were up-regulated, reaching their peak in ICpEC, and then down-regulated between ICpEC and GE. Next, by analyzing the gene expression of *DlBES1* family members after indole-3-acetic acid (IAA) treatment (Figure 9b), the DlBES1 family members responded differently to varying concentrations of IAA. Specifically, *DlBES1.3*, *DlBES1.4*, and *DlBES1.5* up-regulated their expression as the IAA concentration increased from 0 µmol·L^−1^ to 100 µmol·L^−1^, while *DlBES1.7* and *DlBES1.8* down-regulated their expression from 0 µmol·L^−1^ to 50 µmol·L^−1^ and then up-regulated it from 50 µmol·L^−1^ to 100 µmol·L^−1^. Overall, the tested DlBES1 family members exhibited the highest expression at 100 µmol·L^−1^, and all of them showed down-regulated expression from 100 µmol·L^−1^ to 200 µmol·L^−1^, possibly due to the inhibitory effects of excess IAA on the *DlBES1* genes.

## 3. Discussion

Brassinosteroids, a class of plant-specific steroid hormones, exert critical regulatory functions in plant growth, development, and stress responses. Temperature fluctuations, drought, salinity, and insect infestations are among the environmental factors that present formidable challenges to the growth and development of *D*. *longan*. The survival of this plant hinges on intricate molecular mechanisms. Central to the BR signaling pathway is the Bri1 EMS Suppressor 1 (*BES1*) transcription factor family, which modulates downstream gene expression to govern plant growth and stress responses. As observed in other plant species, *BES1* in *D*. *longan* is likely to play a vital role in mediating BR signaling and orchestrating adaptive responses to environmental stressors. Considering the pivotal role of *BES1* genes in the signaling pathways of *D*. *longan*, investigating their expression patterns is of utmost importance. Undertaking extensive bioinformatics research will not only aid in the precise identification of abiotic stress-tolerant genes in *D*. *longan* but also offer significant insights into their regulatory mechanisms, thereby providing a theoretical foundation for the breeding and cultivation management of stress-resistant *D. longan*.

Transcriptional regulation underlies the biological effects of hormones in many plants [54]. The *BES1* gene encodes a transcription factor that plays a key role in the BR signaling transduction pathway [8]. BES1 transcription factors are widely present in plants. Since its identification in *A. thaliana* [51], genome-wide identification of the *BES1* gene family has been conducted in many species, including *Gossypium hirsutum* Linn. [55], *Glycine max* (L.) [56] *Brassica napus* L. [57], *Zea mays* L. [58], *Brassica oleracea* L. [59], *M. pumila* [60], *O. sativa* [52], *P. nigrum* [53], and *Triticum aestivum* L. [61], while few reports have focused on the functions of this gene family in *D. longan*. The structure of a gene is a typical feature of the gene family that represents their evolutionary process [62]. The comprehensive analysis of the phylogeny, the protein motif, and the gene structure of *DlBES1* genes revealed that the genes of the same group were highly conserved for the types of motifs, gene lengths, and even the distribution positions of exons and introns, indicating that the genes within the group may have similar biological functions, consistent with the results of other studies [63]. Phylogenetic trees summarize valuable information about gene evolution and can help us better understand the evolutionary relationships between genes [64].

In this study, a total of 41 *BES1* genes were obtained from six plants: *A. thaliana*, *O. sativa*, *C. lanatus*, *V. vinifera*, *M. pumila,* and *D. longan*. According to the deep duplication nodes of the *BES1* gene family, BES1 can be divided into four gene subfamilies from subfamily I to IV. In terms of the number of *BES1* genes, longan and grape both have eight *BES1* genes, which is more than that of herbs such as *A. thaliana*, *O. sativa*, and *V. vinifera*. However, the number of *BES1* genes in *D. longan* is significantly less than that in *M. pumila*, which is a closely related woody plant. This suggests that the number of *BES1* genes in apples may have increased during the evolution of the *DlBES1* genes. Subfamily I and II contain three-quarters of the BES1 genes, indicating that these two subfamilies, especially subfamily I, maybe the oldest of the four subfamilies just based on the number of BES1 genes. Gene structure analysis showed that *DlBES1.2* and *DlBES1.7* of subfamily I contain the largest number of conserved motifs, while *DlBES1.8* contains a large number of exons, indicating that this subfamily might assume the main function of BES1 genes. Notably, all BES1 family members have motif 1 and motif 2, which suggests that motif 1 and motif 2 are highly conserved and might play an important role in *DlBES1* genes.

The DlBES1 gene family contains a variety of elements that regulate plant growth and respond to environmental stress, which may help plants resist various stresses and regulate their growth. In the gene comparison, the *BES1* genes of longan were more similar to those of *A. thaliana* and far away from that of rice, suggesting that the *BES1* genes of monocots and dicots may be different in plant evolution. The duplication event of a gene is another important feature that represents the evolution of gene families. During the duplication of a gene, its functions gradually become complex and diverse [65]. Segmental duplications of multiple genes occur through polyploidy by chromosome rearrangements [66]. The analysis result of the intraspecific collinearity relationship shows that only *DlBES1.3* in Chr6 and *DlBES1.4* in Chr7 have a collinear relationship, indicating that the ancestors of longan probably had one segment duplication event in their evolutionary history; the results are similar to those of Wang Shiping’s team [67]. Within the collinearity region, those gene pairs that present a conserved state are most likely to show a certain degree of functional similarity. Therefore, we can use the existing research results of model crops to speculate the potential function of the longan BES1 gene. For example, the function of some *BES1* genes in *A. thaliana* in specific physiological processes is known. We can speculate that the known collinear BES1 genes in longan may have similar or related functions, which can be used as a reference for further study of longan *BES1* genes [68].

Promoters contain important cis-acting elements for gene initiation and transcription regulation [69]. The upstream sequences of the BES1 genes have a variety of cis-acting elements (Figure 5), including stress, hormone, and light response elements, indicating its potential involvement in a variety of stress and hormone response processes as a mechanism of promoting plant growth, development, and stress tolerance. Recent reports reveal that specific promoter elements within *BES1* genes are essential for mediating a plant’s response to environmental stresses such as drought, salinity, and temperature fluctuations [70]. These promoter elements are responsible for initiating the transcription of *BES1* genes, thereby influencing the expression levels of BES1 and promoting resistance to drought, salinity, temperature changes, and other stresses.

The BR–auxin interaction mechanism is the regulation of genes involved in auxin response and transport by the BES1 transcription factor [71]. The non-linear alterations in the expression patterns of *DlBES1* genes under IAA treatment hint at a potential feedback regulatory mechanism within the framework of how the BES1 transcription factor modulates the auxin response to suppress the expression of BES1 genes. Investigations into the expression of BES1 genes across varying temperatures have unveiled that the expression trajectories of *DlBES1* gene family members diverge markedly under different temperature and heat stress scenarios. Future endeavors, encompassing gene-specific overexpression studies as well as analyses of BES1 knockout plants, hold significant promise for unraveling the precise roles that these genes play.

Under different temperature conditions, the *DlBES1* gene family members showed specific expression. At low temperatures, the expression DlBES1 genes enhances cell viability by activating low-temperature response genes, involving membrane lipid metabolism, osmotic regulation, and antioxidant defense. Specific members such as *DlBES1.1*, *DlBES1.4*, *DlBES1.5*, and *DlBES1.6* may regulate the degree of membrane lipid unsaturation and cell osmotic adjustment ability, and reduce cold damage. At high temperatures, the expression of *DlBES1* genes is different [72]; they may be involved in the regulation of heat shock protein expression and cell membrane stability, and may reduce the effects of high temperature. This process involves the interaction of temperature sensors, transcription factors, and signaling molecules. *DlBES1* genes regulate the expression of stress-resistant genes to resist temperature damage.

## 4. Materials and Methods

### 4.1. Plant Materials and Transcriptome Data Sources

In this study, we used somatic embryos (SEs) of longan ‘Honghezi (HHZ)’ as the main research material. These materials were carefully cultivated in the Institute of Horticultural Biotechnology, Fujian Agriculture and Forestry University, and obtained according to the methods previously studied by the institute [73,74]. The whole culture process includes three stages: embryogenic callus (EC), incomplete compact embryo culture (IcpEC), and globular embryo (GE). Specifically, we selected 0.2 g of embryogenic callus (EC), placed it in MS medium, and treated it with IAA at concentrations of 50 μM, 100 μM, and 200 μM. In order to ensure the reliability of the experimental results, three biological replicates were performed for each treatment. After the treatment, we collected all of the samples, quickly frozen them in liquid nitrogen, and then stored them in a refrigerator at −80 °C to maintain the stability and activity of the samples and provide protection for subsequent experimental analysis. In this study, the longan BES1 gene sequence, CDS sequence, amino acid sequence, and gene annotation information were downloaded from the longan genome database independently constructed by the institute [75].

### 4.2. Identification of BES1 Genes in D. longan

The reported *A. thaliana BES1* gene sequences were obtained from the TAIR [76] (https://www.arabidopsis.org/, accessed on 23 December 2024) website and homologated to the longan genome database using TBtools-IIv2.154 [77] software. Two methods, the HMM search and bidirectional BLAST comparison, were used to identify longan BES1 family members. Functional domains of candidate proteins were analyzed with SMART website (https://smart.embl.de/), and proteins lacking conserved domains were excluded. The conserved structural domains of the above sequences were viewed using the Interpro [78] (https://www.ebi.ac.uk/interpro/, accessed on 23 December 2024) website, and eight longan *BES1* gene sequences were finally obtained by filtering them according to whether they included the BES1_N structure (PF05687). Referring to the longan genome annotation file and the nomenclature of BES1s from other species, they were sequentially named *DlBES1.1*~*DlBES1.8*. The number of amino acids, relative molecular weight, theoretical isoelectric values, instability index, lipid solubility coefficients, and hydrophilicity of the members of the *DlBES1* genes were analyzed by using the online software Expasy [79] (https://www.expasy.org/, accessed on 23 December 2024), and subcellular localization of the *DlBES1* genes was performed using cello [80] (https://wolfpsort.hgc.jp/, accessed on 23 December 2024) online software.

### 4.3. Evolutionary Relationships, Genomic Architecture, and Conserved Sequence Motif Investigation

Phylogenetic trees of the BES1 family of six species, namely longan, watermelon, apple, grape, rice, and *A. thaliana*, were constructed using MEGA11 [81] software. Grape and rice BES1 sequences were obtained from the website of Phytozome [82] (https://phytozome-next.jgi.doe.gov/, accessed on 23 December 2024), apple BES1 sequences were obtained from NCBI [9], and watermelon BES1 sequences were obtained from CuGenDB [16] (http://cucurbitgenomics.org/, accessed on 23 December 2024) website. The neighbor-joining method was chosen to perform the 1000-bootstrap replication analysis for the above sequences and other parameters were defaulted. Finally, iTOL (https://itol.embl.de/, accessed on 23 December 2024) [83] online software was used to visualize the above results. The longan BES1 sequences were imported into MEME [84] (https://meme-suite.org/meme/, accessed on 23 December 2024) online software and the number of motifs was set to 10 to predict and analyze the above sequences. The NCBI [9] (https://www.ncbi.nlm.nih.gov/, accessed on 23 December 2024) online website was used to analyze the above sequences using the Batch CD search function of the NCBI [9] online website in terms of the structural domains contained in the amino acid sequences of longan BES1; the longan BES1 gene annotation file was imported into TBtools-IIv2.154 [77] software, and the results of the above three types of analyses based on the Gene StructureView function of TBtools software were visualized. Finally, the longan BES1 motif, intron, and conserved structure visualization maps were obtained.

### 4.4. Chromosomal Positioning, Cis-Regulatory Element Examination, and Synteny Study

Longan *BES1* gene annotation files were imported into TBtools software to obtain the chromosomal location, cis-acting elements analyses, and collinear analysis with other species. PlantCARE [85] (https://bioinformatics.psb.ugent.be/webtools/plantcare/html/, accessed on 23 December 2024) was used to analyze the 2000 bp upstream sequence of longan *BES1* for cis-acting element analysis. Stress response elements, hormone response elements, plant growth and development elements, and light response elements as were used as horizontal coordinates, the longan BES1 genes served as vertical coordinates, and a two-dimensional heat map was plotted. *A. thaliana* and rice genome-wide annotation information was downloaded from the TAIR website and the Phytozome [82] (https://phytozome.jgi.doe.gov/, accessed on 23 December 2024) website, respectively, and the genome-wide annotation information was analyzed using MCscanX (https://github.com/wyp1125/MCScanX/, accessed on 23 December 2024) [86] to analyze the collinear relationship between the above species and longan. Finally, TBtools was used to draw and enhance the visualized images of the gene collinear relationship between longan, *A. thaliana,* and rice species. 

### 4.5. Hormonal Processing of D. longan EC, RNA Extraction, and qRT-PCR Analysis

Longan EC materials with good growth status were selected, mixed thoroughly, and added to 100 μmol-L-1 IAA liquid medium in equal amounts. Longan EC materials added to MS liquid medium without any hormone were used as a blank control. The above treatments were set up in three replicates, and longan EC materials were cultured in darkness at 25 °C for 24 h. After that, they were filter-dried and frozen in the refrigerator at −80 °C for later use. DNAMAN6 software was used to design the qRT-PCR primers for longan *BES1* genes, and the primer sequences are shown in Table S1. The RNA of the above hormone-treated materials was extracted using the TransZol kit (TransGen Biotech, Beijing, China), and the specific method was described in the instruction manual. The extracted RNA was reverse-transcribed into cDNA using the Revertaid Master Mix (Thermo Fisher Scientific, Beijing, China) kit. Ubiquitin [87] (UBQ) was used as an internal reference. The Roche Light Cycler 96 real-time fluorescence quantitative PCR instrument (Roche Diagnostics, Basel, Switzerland) was used to detect the *BES1* gene expression levels after IAA hormone treatment at the longan EC stage.

### 4.6. Analysis of Specific Expression of DlBES1 Family Genes

The FPKMs (Fragments Per Kilobase Million) of longan *BES1* gene members at three stages of early somatic embryogenesis (EC, ICpEC, GE), under high and low temperatures (35 °C, 15 °C), with 2,4-D treatments for different days, and at nine different tissue sites (seeds, roots, stems, leaves, flowers, buds, fruit pulp, young fruits, and pericarp) were obtained from the longan early somatic embryogenesis transcriptome database (SRA050205). The above data were log-transformed (log2) and finally plotted into a heat map using TBtools.

## 5. Conclusions

In this study, eight *BES1* genes were identified in *D*. *longan* and their functional characteristics were analyzed. All DlBES1 proteins were localized in the nucleus, and some DlBES1 proteins were also present in the cytoplasm. Through the analysis of cis-acting elements, we found that *DlBES1* genes have many cis-acting elements related to hormone response and environmental stress tolerance, indicating that *DlBES1* genes may promote plant growth and development through hormone response and enhance environmental stress tolerance. *DlBES1* genes showed different expression patterns in nine tissues, indicating that the *BES1* genes have a variety of functional roles in promoting root development, stem elongation, leaf growth, and flower and fruit ripening. The differential expression of *DlBES1* in hormone response experiments under IAA treatment indicates that there may be an unknown feedback mechanism in the regulation of BES1 response to auxin. Our preliminary results provide a solid foundation for further study of the role of the *DlBES1* genes in longan. It provides new insights into the functional characteristics of the *BES1* genes in longan and has certain significance for revealing the mechanism of the *BES1* genes in plant hormone signal transduction and stress response.

## Figures and Tables

**Table 1:** Detailed information of 8 *DlBES1* genes and their encoded proteins.

Gene Accession	Gene ID	Size/aa1	MW2/Da	Theoretical pI3	Instability Index	Aliphatic Index	GRAVY4	Subcellular Localization
Dlo009294	DlBES1.1	328	35,264.37	8.79	56.42	55.09	−0.584	Nuclear
Dlo012787	DlBES1.2	701	78,532.89	5.44	41.64	75.35	−0.373	Cytoplasmic and Nuclear
Dlo013572	DlBES1.3	309	33,848.83	8.90	62.70	56.67	−0.708	Nuclear
Dlo015717	DlBES1.4	327	35,229.57	8.83	65.83	65.96	−0.509	Nuclear
Dlo021810	DlBES1.5	327	34,990.97	8.59	62.87	54.98	−0.606	Nuclear
Dlo022049	DlBES1.6	202	21,720.31	9.62	62.33	60.89	−0.669	Nuclear
Dlo028252	DlBES1.7	642	72,341.40	5.48	36.96	75.34	−0.366	Cytoplasmic and Nuclear
Dlo028973	DlBES1.8	1178	130,421.60	9.04	43.79	75.98	−0.501	Nuclear

Note: aa1: amino acid number; MW2: molecular weight; pI3: isoelectric point; GRAVY4: grand average of hydropathicity.

## Data Availability

Publicly available datasets were analyzed in this study.

## Supplementary Materials

The supporting information can be downloaded at: https://www.mdpi.com/article/10.3390/ijms26073003/s1.
